# Synthesis and Characterization of Poly(lactic acid) Composites with Organosolv Lignin

**DOI:** 10.3390/molecules27238143

**Published:** 2022-11-23

**Authors:** Zoi Terzopoulou, Eleftheria Xanthopoulou, Nikolaos Pardalis, Christina P. Pappa, Stylianos Torofias, Konstantinos S. Triantafyllidis, Dimitrios N. Bikiaris

**Affiliations:** 1 Laboratory of Chemistry and Technology of Polymers and Dyes, Department of Chemistry, Aristotle University of Thessaloniki, GR54124 Thessaloniki, Greece; 2 Laboratory of Chemical and Environmental Technology, Department of Chemistry, Aristotle University of Thessaloniki, GR54124 Thessaloniki, Greece

**Keywords:** lignin, ball milling, poly(lactic acid), polymer composites

## Abstract

Lignin, being one of the main structural components of lignocellulosic biomass, is considered the most abundant natural source of phenolics and aromatics. Efforts for its valorisation were recently explored as it is mostly treated as waste from heat/energy production via combustion. Among them, polymer-based lignin composites are a promising approach to both valorise lignin and to fine tune the properties of polymers. In this work, organosolv lignin, from beech wood, was used as fillers in a poly (lactic acid) (PLA) matrix. The PLA/lignin composites were prepared using melt mixing of masterbatches with neat PLA in three different lignin contents: 0.5, 1.0 and 2.5 wt%. Lignin was used as-isolated, via the organosolv biomass pretreatment/fractionation process and after 8 h of ball milling. The composites were characterised with Scanning Electron Microscopy (SEM), Fourier Transform Infrared Spectroscopy (FTIR) spectroscopy, X-ray Diffraction (XRD), and Differential Scanning Calorimetry (DSC). Additionally, their antioxidant activity was assessed with the 2,2-Diphenyil-1-picrylhydrazyl (DPPH) method, the colour was measured with a colorimeter and the mechanical properties were evaluated with tensile testing. Ball milling, at least under the conditions applied in this study, did not induce a further substantial decrease in the already relatively small organosolv lignin primary particles of ~1 μm. All the produced PLA/lignin composites had a uniform dispersion of lignin. Compression-moulded films were successfully prepared, and they were coloured brown, with ball-milled lignin, giving a slightly lighter colour in comparison with the as-received lignin. Hydrogen bonding was detected between the components of the composites, and crystallization of the PLA was suppressed by both lignin, with the suppression being less pronounced by the ball-milled lignin. All composites showed a significantly improved antioxidant activity, and their mechanical properties were maintained for filler content 1 wt%.

## 1. Introduction

An integral part of the establishment of a bioeconomy in Europe is the development of biobased plastics, i.e., plastics made from renewable sources, as this will contribute towards the gradual independence of our society from finite and non-renewable fossil sources. Lignocellulosic biomass is an abundant source of high added value chemicals. Lignin is one of the three main components of lignocellulose, with a content of ca. 10–25%, depending on the nature of the biomass, with the other two being cellulose (30–50%) and hemicellulose (15–35%) [1]. The utilization of lignin is a topic of intense research in the last decade [2], and one of the simplest approaches is its use as a filler in polymer composites [3,4,5,6]. Lignin can reduce the cost and increase the biobased nature of polymer composites, but its polar character, owing to the surface hydroxyls and carboxyls, often causes a lack of interfacial interaction with hydrophobic/non-polar polymers, such as PE or PP, which leads to a bad dispersion and therefore poor mechanical properties. On the other hand, polymers with oxygen backbone and terminal functionalities, such as epoxy and phenol-formaldehyde resins, PLA, and others, which may exhibit enhanced chemical interaction with lignin. Another parameter, that may affect the dispersion of lignin in the bulk polymer, is the inherent size of the as-isolated technical lignins, which can be higher than ca. 10 μm [7]. Furthermore, π–π interactions of the phenolic units of lignin with aromatic-based polymers may also enhance the chemical bonding and subsequently improve the properties of the formed composite. Another common approach to further increase the compatibility of a lignin with a polymer matrix is its tailored functionalization (e.g., by acetylation, and oxypropylation) [8,9]. While functionalization can help retain the mechanical properties in the polymer/lignin composites, it may negatively impact the UV blocking and antioxidant properties of the lignin [10]. Additionally, the effect of the functionalization is often minimal and only required in larger lignin contents (>10 wt%).

Reducing the size of fillers is known to be beneficial for polymer composites, as larger filler surface areas can impart improved properties to the polymeric matrices [11]. For this reason, methods to reduce the particle size of the lignin have been in the spotlight in order to enable the introduction of a lignin, as a polymer additive, easier and more beneficial [7,12]. Ball milling is a simple and readily available method that can be used in wet or dry conditions to substantially reduce the particle size of the lignin [13].

In previous work, by our group, kraft lignin was added to a PLA matrix, via melt mixing, and the composites had both antioxidant and antibacterial activity, an increased hardness and Young’s modulus, accelerated crystallization, and a slightly improved thermal stability [14,15,16]. In this work, another type of biorefinery lignin, the so-called organosolv lignin, was evaluated as a filler in PLA. In brief, the organosolv lignin can be isolated from various types of lignocellulosic biomass by treating the biomass in alcohol (ca. ethanol–water mixtures), at 170–200 °C, under hydrothermal and autogeneous pressure conditions; small amounts of sulfuric acid is sometimes used to facilitate the disruption of the recalcitrant lignocellulose structures and to liberate a larger fraction of lignin in the liquid stream of the process [17,18]. The effect of ball milling of the organosolv lignin, on the properties of the PLA matrix, was also evaluated. Since large loadings of lignin can reduce the tensile strength of the PLA, the effect of smaller loadings, up to 2.5 wt%, were chosen in this study. The composites were prepared with a two-step approach: first the PLA/lignin masterbatches were prepared by solution mixing, followed by melt blending. This approach was examined as an easy and scalable process in order to prepare the PLA-based composites with adequate properties, containing lignin that is not chemically functionalized.

## 2. Results and Discussion

### 2.1. Characterization of Lignin

**Figure 1:**
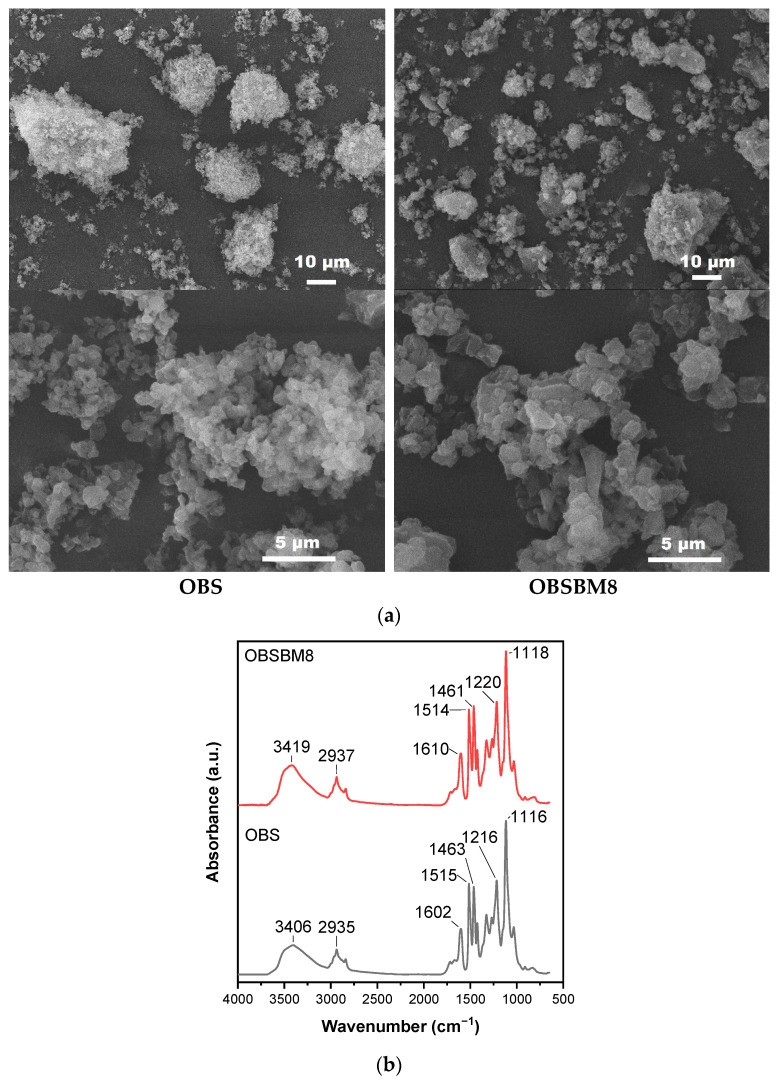
Characterization of lignin (**a**) SEM images, (**b**) FTIR spectra.

The particles of technical lignins that are available on the market can range from 10 μm to >100 μm [7]. The morphology of the isolated organosolv lignin, in the present study, before (OBS) and after ball milling (OBSBM8), is shown in the SEM images of Figure 1a. The parent organosolv lignin is already comprised of relatively small primary particles of different shapes in the submicron scale, with an average of ~1 μm, which tend to aggregate towards larger formulations of ca. 10–50 μm. The use of ball milling led to some breaking of these aggregates, but it had a small effect on the size of the primary particles, which remained about ~1–2 μm.

The FTIR spectra of OBS and OBSBM8 are shown in Figure 1b. The broad band at ~3400 cm^−1^ is attributed to the O-H bending vibrations of the hydroxyl groups, the band at 2935 cm^−1^ to C-H bending, at 1600 cm^−1^, 1500 cm^−1^, and 1460 cm^−1^ to skeletal bending vibrations of the aromatic rings The broad band at 1220 cm^−1^ is associated with C-C, C-O and C=O stretching of guaiacyl, and at 1115 cm^−1^ to the aromatic C-H in-plane deformation of syringyl units [19]. As can be seen in the FTIR spectra of Figure 1b, no significant difference can be identified between the two lignins, thus suggesting that the structure of OBSBM8 is not affected by the ball milling treatment. The slight increase in the intensity at the peak of 3400 cm^−1^ could be attributed either to a slightly higher H_2_O content (humidity) in the OBSBM8 sample and/or to the marginally higher content of the OH groups in OBSBM8 when compared to the initial OBS (5.27 vs. 5.05 mmol OH/g), as determined by the ^13^P NMR analysis (data shown in Figure S1, Supporting Information and listed in Table S1).

### 2.2. Morphology of PLA/Lignin Composites

**Figure 2:**
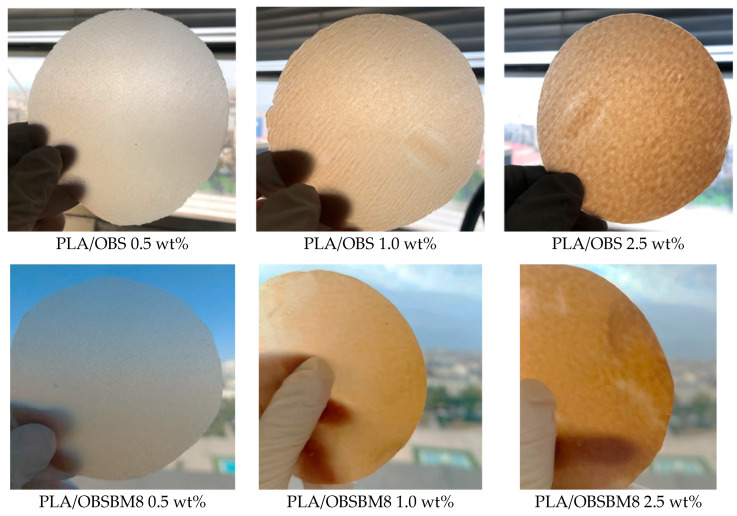
Photographs of the PLA/lignin films.

The visual appearance of the films prepared by compression moulding (Figure 2) shows a macroscopically uniform distribution of the lignin in the PLA matrix without any visible particles or aggregates. In contrast, the PLA/kraft lignin composites, prepared without the use of masterbatch directly in the melt compounder, resulted in a visibly heterogenous polymer [14].

**Figure 3:**
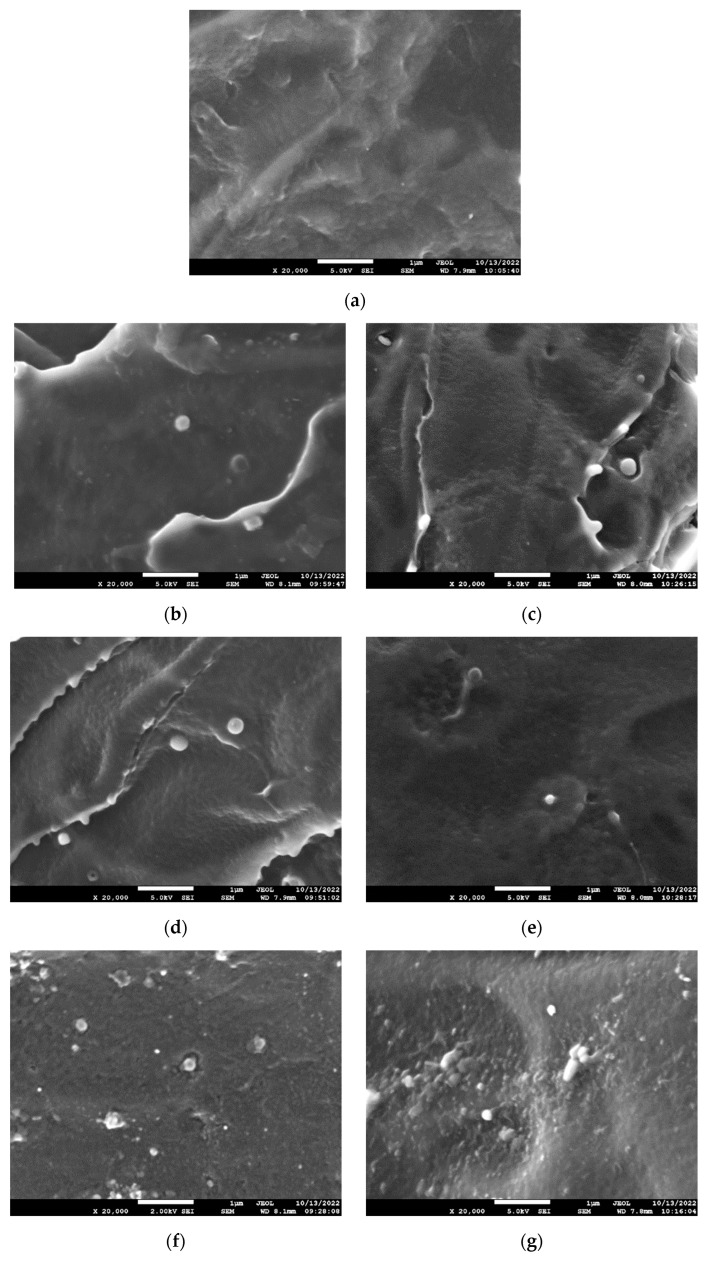
SEM micrographs of cryo-fractures cross-sections of PLA and its composites with OBS and OBSBM8 lignin. Magnification ×20,000. (**a**) PLA neat, (**b**) PLA + 0.5% OBS, (**c**) PLA + 0.5% OBSBM8, (**d**) PLA + 1% OBS, (**e**) PLA + 1% OBSBM8, (**f**) PLA + 2.5% OBS, (**g**) PLA + 2.5% OBSBM8.

The cross-sections of the films were observed using SEM (Figure 3), and it had a homogenous morphology with no visible defects or aggregates in the content up to 1 wt%, confirming a good dispersion of the lignin. The lack of aggregates might have resulted from the sonication treatment during the composite preparation. As the lignin content increased, more small particles were observed, e.g., Figure 3g, where several bigger particles were also detected, which could be clusters of individual particles. However, debonding, which is an indication of a weak interface, is not detected. Such debonding was visualised by SEM in the PLA composites containing more than 5 wt% of lignin [20].

**Figure 4:**
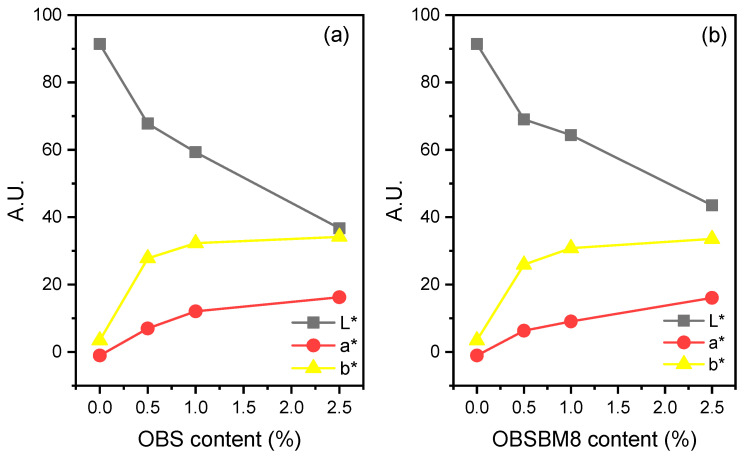
CIE L*a*b* Coordinate of PLA and its composites with (**a**) OBS lignin, (**b**) OBSBM8 lignin. L*: perceptual lightness, a* and b*: red–green and blue–yellow.

To quantify the colouration of the PLA films after the incorporation of the lignin, the colour was measured with a colorimeter, and it is expressed with CIE coordinates in Figure 4. As a white-coloured material, neat PLA has a L* value of 91.4 and a* and b* values of almost zero. After the introduction of both lignins into the PLA, L* progressively decreased while a* and b* increased, meaning that the films became darker, redder, and yellower as expected due to their brown colour (Figure 2). When comparing the two sets of composites, the decrease in L* and the increase in a* and b* are almost similar, or slightly bigger for the PLA/OBS when compared to the composites prepared with the ball-milled lignin. This is in accordance with the similar size of the lignin particles, before and after milling, as light scattering is affected by the filler particle size.

The reflectance of PLA was measured at 61% and it significantly decreased in the presence of lignin. In particular, films of PLA with 0.5, 1, and 2.5% OBS lignin had reflectance values 9.3, 5.2 and 2.9%, respectively, and films with 0.5, 1, and 2.5% OBSBM8 lignin had reflectance values 11.4, 7.3 and 3.5%, respectively. Thus, the addition of the lignin turns the PLA from being highly reflective surface to a diffusing surface, a feature desired in polymers for outdoor use where carbon black is commonly used as UV stabilizers due to their ability to backscatter light. It was also shown, previously, that the lignin can strongly improve the UV barrier property of the PLA due to the absorption of UV light by the conjugated phenolic crosslinks [10,21,22,23].

### 2.3. FTIR Spectroscopy

**Figure 5:**
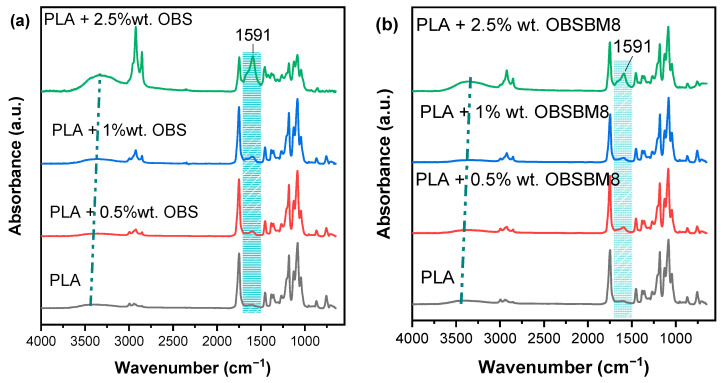
FTIR spectra of (**a**) PLA/OBS and (**b**) PLA/OBSBM8 composites.

The FTIR spectra of PLA and its composites with organosolv lignin are shown in Figure 5.

The main IR bands of the PLA are at 1748 cm^−1^, 1453 cm^−1^, 1180 cm^−1^ and 1085 cm^−1^, which correspond to C=O stretching, -CH_3_ bending, and C-O-C stretching. A small band, assigned to hydroxyl groups, is also detectable at ~3450 cm^−1^. In all composites, a new band at 1591 cm^−1^ emerged, which can be associated with the C-H bending of the aromatic rings of lignin [24,25], and its intensity increased with the increasing lignin content. Additionally, the hydroxyl band of the PLA increased in intensity, with the hydroxyl groups of the lignin also contributing to this peak, which shifted towards smaller wavenumbers in the composites; this is an indication that hydrogen bonding occurred between the terminal hydroxyl groups of the PLA and the functional groups of the lignin. It should be mentioned that the increase in the intensity of both these peaks, in the system with 2.5 wt% of OBS, in comparison with the corresponding system of OBSBM8, may be assigned to the interaction between the filler and the matrix. Specifically, the higher the value of -OH content in the OBSBM8 lignin can improve its interaction with the PLA, leading to the decrease in free -OH groups in comparison to the PLA/OBS composites.

### 2.4. Thermal Properties and Crystallinity

The thermal behaviour of the PLA/lignin composites was studied with DSC. The resulting graphs are shown in Figures S2 and S3, and all samples show a glass transition, cold crystallization, and subsequent melting. No significant crystallization was detected during cooling. The thermal characteristics, i.e., glass transition temperature (*T*_g_), cold crystallization temperature (*T*_cc_), melting temperature ™ and crystallinity (*X_c_*) are summarized in Table 1. 

**Figure 6:**
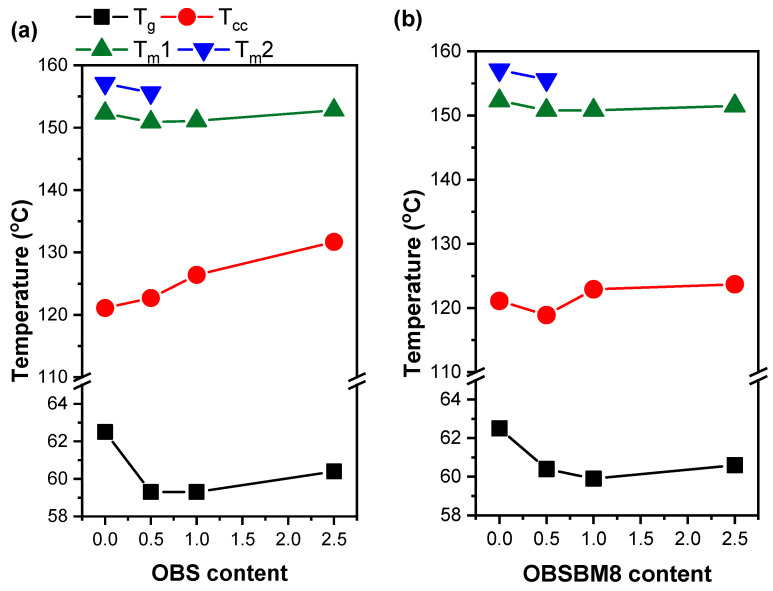
Effect of (**a**) OBS lignin and (**b**) OBSM8 lignin content on the *T*_g_, *T*_cc_ and *T*_m_ of PLA.

In Figure 6, the effect of the lignin type and content on the thermal transitions of the PLA is presented. The addition of lignin slightly decreases the *T*_g_ and *T*_m_ and increases the *T*_cc_ of the PLA. The composites also crystallize less, as the *X_c_* is smaller and decreases with an increased lignin content. The increase in *T*_cc_ and the suppression of crystallinity are attributed to a reduced PLA chain mobility, caused by the interactions of the PLA with the lignin and its ability to absorb heat during heating, which prevents it from being absorbed by the PLA [21,26,27,28,29]. The small decrease in the *T*_g_ was observed before [7,26,30,31,32,33] and it was attributed to the beneficial interactions between the lignin and the PLA, possibly by a plasticizing effect of a low molecular weight fraction of the lignin. The formation of less perfected crystals could have caused the slight reduction in the *T*_m_. In the PLA neat and the PLA + 0.5% wt. samples, the double melting peak is associated with recrystallization and the melting of perfected crystals, which does not occur in higher lignin loadings due to the restricted chain mobility. When comparing the effect of lignin type to the thermal transitions, it is obvious that the increase in *T*_cc_ is limited in the case of OBSBM8. This is related to the smaller size of the aggregates after ball milling, which in turn limits the reduction in chain mobility.

**Figure 7:**
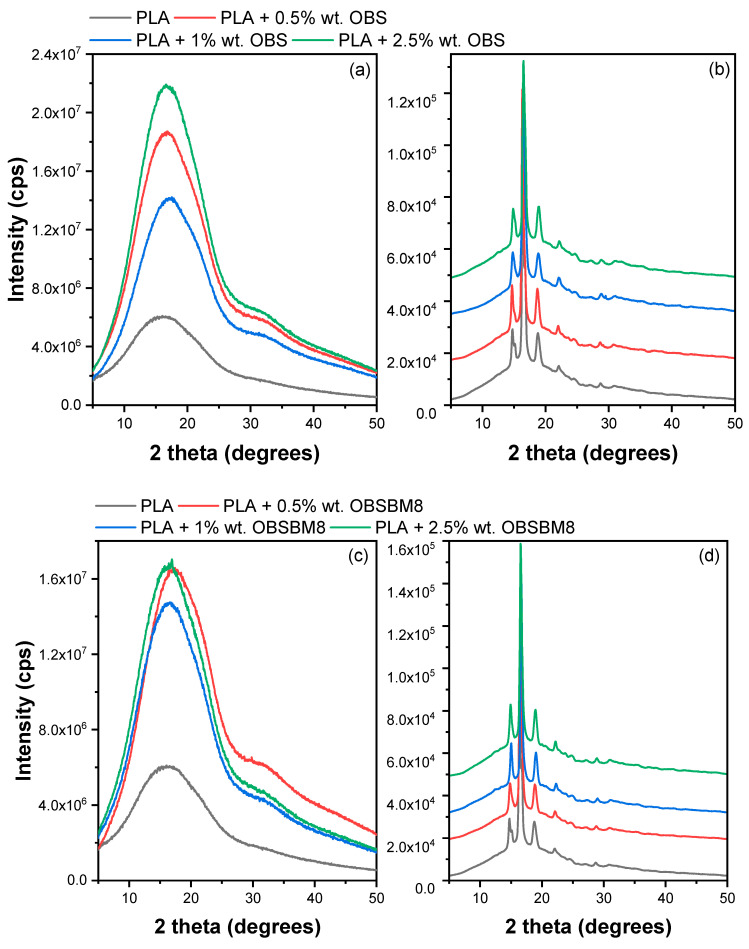
XRD patterns of (**a**,**b**) PLA/OBS and (**c**,**d**) PLA/OBSBM8 samples after quenching and after annealing at 110 °C for 1 h.

XRD patterns of both amorphous films and after annealing at 110 °C for 1 h were recorded. The patterns are shown in Figure 7. Before annealing, the PLA is received amorphous with a typical halo pattern. After annealing, both series of composites show the expected crystalline diffraction peaks at 2θ = 14.8°, 16.5°, 19° and 22°, which correspond to the crystal planes (010), (200/110), (203) and (210). The position of the peaks is not altered by the presence of the lignin, so the crystal structure of the PLA is the same in all samples. The % crystallinity was calculated with Equation (2), which is included in Table 1. It is worth mentioning that while the crystallinity, calculated from Equation (1), using the DSC data subtracts the enthalpy of cold crystallization and corresponds to the crystallinity of the material before heating in the DSC chamber, the crystallinity, calculated from XRD, corresponds to the cold crystallization that took place during the annealing of the amorphous films. Thus, it is expected that the two values will differ significantly. Indeed, neat PLA has *X_c_* = 35%, and it is reduced in the presence of all fillers, except for 0.5% wt. OBS, where it remains unaffected. In all, this trend agrees with the trend observed in DSC, where a suppression of crystallinity was also deducted.

### 2.5. Antioxidant Activity

**Figure 8:**
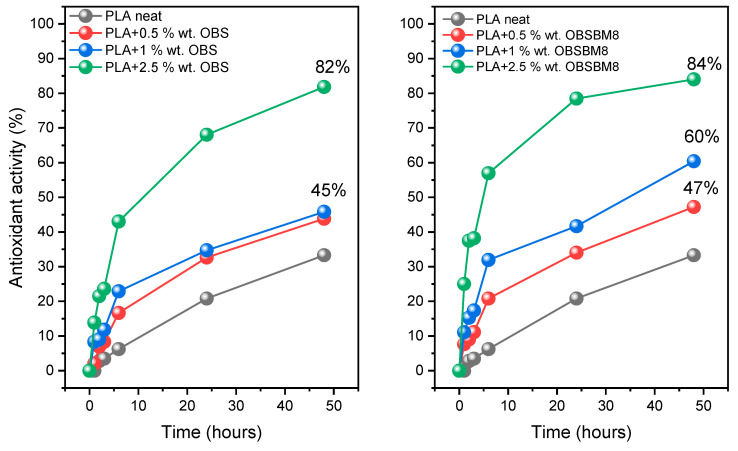
Effect of time, size, and concentration of lignin on the antioxidant activity of PLA.

The radical scavenging ability of the PLA/lignin composites was assessed with the DPPH method. In Figure 8, the effect of the lignin type and content on the antioxidant activity of the PLA is presented.

The presence of both OBS and OBSBM8 lignin strongly enhanced the DPPH scavenging activity, which as expected, increased with the increasing lignin content and time, reaching 84% with only 2.5 wt% of OBSBM8 after 48 h. During the first 6 h, a steep increase in the antioxidant activity was observed, followed by a more gradual increase until the 48 h mark. Overall, the composites with OBSBM8 resulted in slightly higher values of antioxidant activity in comparison with OBS. These results are in agreement with the previous studies, as it has been found that a reduction in the particle sizes of the filler, enhances the antioxidant activity of the final composites [34]. Furthermore, the PLA/OBSBM8 system, demonstrated an excellent radical scavenging using small amounts of unmodified lignin, which has a big advantage when compared to similar studies where modification of the lignin and/or a high percentage of the filler was used [10,32,35].

### 2.6. Tensile Properties

**Figure 9:**
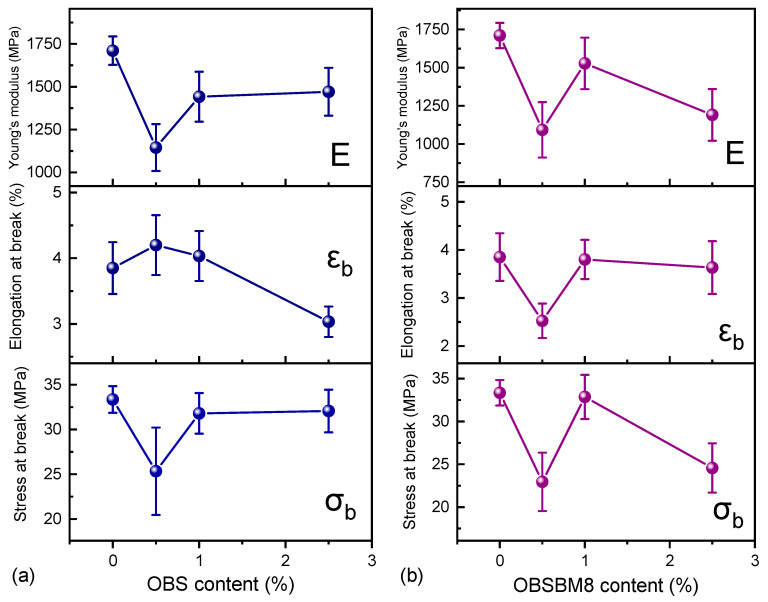
Effect of (**a**) OBS lignin and (**b**) OBSBM8 lignin on the tensile characteristics of PLA. The lines are drawn to guide the eye.

One of the main challenges of composites with lignin is maintaining the mechanical properties of the polymeric matrix. Tensile tests were performed and the resulting values of tensile stress at break (σ_b_), elongation at break (ε_b_), and Young’s modulus (E) are reported in Figure 9, while indicative stress–strain curves are shown in Figure S4. Neat PLA has a σ_b_ = 33.4 ± 1.5 MPa, ε_b_ = 3.9 ± 0.5% and E = 1710 ± 83 MPa. With the addition of either type of 0.5 wt% lignin, σ_b_ and E decrease, and ε_b_ remains constant for OBS and decreases for OBSBM8. This low fraction of lignin could be insufficient for transferring stress from the filler to the matrix and vice versa, resulting in premature failure. Increasing the lignin content to 1 wt% yields composites with a still decreased E, but both σ_b_ and ε_b_ remain almost unaffected. At 2.5 wt%, OBS reduces εb and maintains its σ_b_, while OBSBM8 reduces the σ_b_ and maintains the ε_b_. Thus, the effect of lignin size is more prominent as its content increases. Overall, it can be deduced that at 1 wt% of both lignins the mechanical properties of the PLA are practically consistent.

Tensile tests have also been performed in previous works concerning PLA/lignin composite materials. Different types of lignin’s modifications have been applied to adjust and/or enhance the mechanical properties of the final materials. Specifically, the acetylation of lignin was proven to reduce the size of the aggregates, leading to only slightly improved tensile values [22,25,28,36], while its modification with methacrylates resulted in composite materials with an increasing elongation at break [23,31]. Furthermore, the reduction in the size of the particles after ball milling, with surfactants, seemed to positively affect the mechanical properties of the final materials [21,37]. On the other hand, the use of deep eutectic solvents exhibited a drop in the tensile features of the PLA [26]. Thus, the presented data is in accordance with the literature, showing the advantages of using low percentages of unmodified lignin and avoiding additional energy and time-consuming procedures.

## 3. Materials and Methods

### 3.1. Materials

The poly (lactid acid) used was Ingeo^TM^ Biopolymer 3052D (NatureWorks) and it was kindly donated by Plastika Kritis S.A., Greece, which is designed for injection moulding. It contains ~96% of L- and ~4% of D-lactide and has Mn = 75,300 g/mol. The organosolv lignin (OBS) and OBSBM8 (OBS after 8 h of ball milling) were prepared at the Laboratory of Chemical and Environmental Technology, Department of Chemistry, AUTh. All other solvents and materials used were of analytical grade.

### 3.2. Isolation and Characterization of Lignin

In a typical experiment, beech wood sawdust was hydrothermally treated with an ethanol–water mixture in an autoclave, stirred in a high-pressure reactor using 1.9% *w*/*w* (of biomass) with aqueous H_2_SO_4_ as the acidic catalyst, at 175 °C for 60 min. At the end of the treatment, the solid biomass was separated from the liquid by filtration, and the solubilized lignin was recovered from the liquid by precipitation (more details can be found in a previous report [18]. The solid lignin was dried at 80 °C under a vacuum for 6 h. Ball-milled lignin (OBSBM8) was prepared in a ball mill/planetary mill ( (FRITSCH, Idar-Oberstein, Germany) by grinding for 8 h at 400 rpm.

### 3.3. Preparation of PLA-Lignin Composites

#### 3.3.1. Masterbatch Production via Solution Casting

The PLA-lignin masterbatches were prepared using the solution casting technique. In brief, 2 g of PLA were dissolved in 20 mL of chloroform (10% *w*/*v*). Subsequently, the quantity of lignin for a final concentration of 0.5 wt%, 1 wt% and 2.5 wt% in 10 g of PLA/lignin nanocomposites was diluted in acetone (3% *w*/*v*) and were dispersed in a sonic bath for 1 h to achieve complete dissolution. The two different solutions were mixed under magnetic stirring until the elimination of the quantity of the included solvents, which were then placed in Petri dishes overnight.

#### 3.3.2. Fabrication of PLA/Lignin Nanocomposites via Melt Mixing

The PLA-based composites, reinforced with lignin, were prepared by melt-mixing. Prior to that, the PLA, and the PLA-lignin masterbatch films were dried overnight, under vacuum, at 110 °C. To obtain composites, each masterbatch film and the remaining amount of the dried PLA were introduced into a twin screw co-rotating extruder, operating at 190 °C and 30 rpm for 15 min. In total, six composites with the two different types of lignin (parent and ball-milled) at different contents, 0.5, 1, and 2.5% wt., were prepared. The composition of the materials is shown in detail in Table 2.

Films of each sample were prepared by compression moulding in a thermopress at 180 °C. The films had a thickness of 250 ± 25 μm. After melt pressing, the films were cooled rapidly at room temperature. Half of the films prepared were annealed at 110 °C for 1 h.

### 3.4. Characterization

#### 3.4.1. Scanning Electron Microscopy (SEM)

The surface of the films was studied using a JEOL JMS 7610 F (Jeol, Freising, Germany) scanning electron microscope, operating at 10 kV and equipped with an energy dispersive X-ray (EDX) Oxford ISIS 300 micro-analytical system.

#### 3.4.2. Colour Measurements

Colour measurements were performed using a Datacolor Spectraflash SF600 plus CT UV reflectance colorimeter (Datacolor, Marl, Germany) using the D65 illuminant, 10° standard observer with UV component excluded and specular component included. In each case, five-fold measurements were performed using a special holder (Datacolor) and the mean values were calculated. The colour values were calculated using the CIE L*a*b* colour space system. In this system, L* represents the lightness (L* = 0, black, L* = 100, white). The a* value corresponds to the green–red axis, where negative a* values indicate green and positive a* values indicate red hues. The b* value represents the blue–yellow axis, where negative b* values indicate blue and positive b* values indicate yellow hues.

#### 3.4.3. Fourier-Transformed Infra-Red Spectroscopy (FTIR)

The FTIR spectra of the lignins used, and the produced materials were obtained by FTIR-2000 (Perkin Elmer, Waltham, MA, USA). All spectra were collected in the range from 4000 to 500 cm^−1^ using a resolution of 4 cm^−1^ and 32 co-added scans. The presented spectra were further baseline corrected, normalized, and converted into an absorbance mode.

#### 3.4.4. Differential Scanning Calorimetry (DSC)

Differential scanning calorimetry (DSC) analysis was performed using a PerkinElmer Pyris Diamond DSC differential scanning calorimeter (PerkinElmer, Solingen, Germany) calibrated with pure indium and zinc standards. The system included a PerkinElmer Intracooler 2 (PerkinElmer, Solingen, Germany) cooling accessory. Samples of 5 ± 0.1 mg sealed in aluminium pans were used to test the thermal behaviour of the polymers.

First, the rapidly cooled and annealed films were subjected to heating, with a rate of 20 °C/min, and their crystallinity *X_c_*% was calculated using the Equation (1):

(1) $${X_{c}}_{\;}(\%)=\left(\frac{\Delta H_{m}-\Delta H_{\mathrm{cc}}}{\Delta H_{f}^{0}-\frac{1-\mathrm{wt}\%\;\mathrm{lignin}}{100}}\right)\times 100$$

where Δ*H*_m_, $\Delta H_{\mathrm{cc}}$, $\Delta H_{f}^{0}$ are the experimental melting enthalpy, cold-crystallization enthalpy, and the theoretical heat of fusion of 100% crystalline PLA ($\Delta H_{f}^{0}=$ 93 J/g), respectively.

After the 1st heating scan, the samples were quenched at a rate of 50 °C/min, and re-heated to 20 °C/min to obtain the glass transition (*T*_g_) and cold crystallization (*T*_cc_) temperatures.

#### 3.4.5. X-ray Diffraction (XRD)

XRD was employed to study the semi-crystalline structure of all samples at RT that had previously melted and subsequently fully annealed (110 °C, 1 h). The XRD spectra were recorded by means of a MiniFlex II XRD system (Rigaku Co., Tokyo, Japan), with Cu Ka radiation (0.154 nm), over the 2θ range from 5° to 50° with a scanning rate of 1°/min. The % crystallinity was calculated from the XRD graphs using the Equation (2) of Hay et al. [38]:

(2) $$X_{c}={\left(1+\frac{A_{am}}{A_{c}}\right)}^{-1}$$

where *A_am_* is the area of the amorphous halo and *A_c_* is the area of the crystalline peaks.

#### 3.4.6. Antioxidant Activity

The antioxidant activity of the samples was determined with the 2,2-Diphenyil-1-picrylhydrazyl (DPPH) method, which was developed according to Blois in 1958 [39]. Each film of 0.5 × 1 cm was added to 3 mL of a 5 × 10^−3^ mg/mL DPPH/EtOH solution. The reference sample composed of 1 mL EtOH and 3 mL of the DPPH/EtOH solution.

The samples remained in the solution for 1, 2, 3, 6, 24, and 48 h and the absorbance of each solution was recorded with the aid of a UV-Vis spectrometer (UV Probe 1650, Shimadzu, Tokyo, Japan) at 515–517 nm. The free radical scavenging activity was calculated as reported by Brand et al. according to the following Equation (3):

(3) $$\mathrm{Free}\;\mathrm{Radical}\;\mathrm{scavenging}\;\mathrm{activity}\;(\%)=\frac{Absorbance\;of\;control-absorbance\;of\;solution}{Absorbance\;of\;control}\times 100$$

#### 3.4.7. Tensile Testing

Tensile tests were performed using an Instron 3344 dynamometer, in accordance with ASTM D638 using a crosshead speed of 5 mm/min. The dumb-bell shaped tensile test specimens (central portions 5 × 0.5 mm thick, 22 mm gauge length) were cut in a Wallace cutting press. At least five measurements were performed for each sample, and the results were averaged to obtain the mean values of Young’s modulus, tensile strength at yield and breakpoint, and elongation at break.

## 4. Conclusions

In this work, organosolv lignin, both as-isolated from the pretreatment/fractionation of lignocellulosic biomass and after ball-milling, without any additional chemical functionalization, was used as a filler in the PLA. The as-received organosolv lignin primary particles were ~1 μm, which also formed larger aggregates, while ball milling, at least under the conditions applied in this study, did not induce any further decreases in the size of the already relatively small particles. The composites were prepared with a two-step approach: masterbatches were prepared with solution casting, followed by melt mixing. The PLA/lignin composites were compression moulded into thin films with a brown colour. A good dispersion and hydrogen bonding was detected between the PLA and the lignin. The use of OBSBM8 resulted in better interactions between the filler and the matrix, as shown by FTIR spectra. Crystallization of the PLA was suppressed by both types of lignin. Small amounts of lignin induced an excellent DPPH scavenging activity for PLA and reduced the colouration, making the PLA/lignin composites attractive materials for antioxidant food or medical packaging applications. At 1 wt% of both lignins, the mechanical properties of the PLA were practically consistent. In all, light reflective, antioxidant composites with maintained mechanical properties and containing unfunctionalized micro-sized organosolv lignin, were successfully prepared using a sonication-assisted masterbatch preparation approach.

## Figures and Tables

**Table 1:** Thermal characteristics of PLA and its composites with PLA obtained by DSC (second scan).

Sample	*T*_g_ (°C)	*T*_cc_ (°C)	*T*_m_ (°C)	*X*_c_ (%) ^a^	*X_c_* (%) ^b^
PLA neat	62.5	121.1	152.3, 157.1	10.3	35.0
PLA + 0.5% wt OBS	59.3	122.7	150.9, 155.6	9.5	35.3
PLA + 1% wt OBS	59.3	126.4	151.1	8.1	27.9
PLA + 2.5% wt OBS	60.4	131.7	152.8	6.5	24.5
PLA + 0.5% wt OBSBM8	60.4	118.9	150.8, 155.6	9.8	26.5
PLA + 1% wt OBSBM8	59.9	122.9	150.8	5.6	28.4
PLA + 2.5% wt OBSBM8	60.6	123.7	151.5	5.8	24.4

**^a^** From DSC data, using Equation (1). **^b^** From XRD data, using Equation (2).

**Table 2:** Composition of the materials prepared.

Sample	Masterbatch		Final Nanocomposite
	PLA (g)	Lignin (g)	
PLA neat	-	-	10 g PLA
PLA + 0.5% wt OBS	2	0.05	Masterbatch + 7.95 g PLA
PLA + 1% wt OBS	2	0.1	Masterbatch + 7.9 g PLA
PLA + 2.5% wt OBS	2	0.25	Masterbatch + 7.75 g PLA
PLA + 0.5% wt OBSBM8	2	0.05	Masterbatch + 7.95 g PLA
PLA + 1% wt OBSBM8	2	0.1	Masterbatch + 7.9 g PLA
PLA + 2.5% wt OBSBM8	2	0.25	Masterbatch + 7.75 g PLA

## Data Availability

All the data of this study is included in the manuscript and Supplementary Material.

## Supplementary Materials

The following supporting information can be downloaded at: https://www.mdpi.com/article/10.3390/molecules27238143/s1, Table S1: Quantitative assignments of functional groups and their abundance present in OBS and OBSBM8 lignins, as determined ^31^P-NMR analysis, Figure S1: ^31^P-NMR spectra of OBs and OBSBM8 lignins, Figure S2: DSC scans of PLA/OBS composites, Figure S3: DSC scans of PLA/OBSBM8 composites, Figure S4: Indicative stress–strain curves of PLA and its composites with OBS and OBSBM8 lignin.

## Sample Availability

Samples of the compounds are not available from the authors.
