# Supplementary material for: Synthesis and Characterization of Poly(lactic acid) Composites with Organosolv Lignin
Source: Molecules. 2022 Nov 23;27(23):8143. doi: 10.3390/molecules27238143 (PMC9740318; doi:10.3390/molecules27238143)
Supplement: Supplementary file 1 [file molecules-27-08143-s001.zip › molecules-2004942-final supplementary.pdf]

Electronic Supplementary Information (ESI).

Synthesis and characterization of poly(lactic acid) composites with organosolv lignin

Zoi Terzopoulou[1*], Eleftheria Xanthopoulou[1], Nikolaos Pardalis[1], Christina P. Pappa[2], Stylianos Torofias[2], Konstantinos S. Triantafyllidis[2], Dimitrios N. Bikiaris[1*]

[1] Laboratory of Chemistry and Technology of Polymers and Dyes, Department of Chemistry, Aristotle University of Thessaloniki, GR54124 Thessaloniki, Greece, terzozoi@chem.auth.gr (Z.T.); eleftheria.xanthopoulou@gmail.com (E.X.); npardalis@chem.auth.gr (N.P); dbic@chem.auth.gr (D.N.B.)
[2] Laboratory of Chemical and Environmental Technology, Department of Chemistry, Aristotle University of Thessaloniki, GR54124 Thessaloniki, Greece, christipappa@chem.auth.gr (X.P.); torofias@gmail.com (S.T.); ktrianta@chem.auth.gr (K.T.)
* Correspondence: Z.T. terzozoi@chem.auth.gr; Tel.: +302310997818, D.N.B. dbic@chem.auth.gr; Tel.: +302310997812

I. Methods

Quantitative ^{31}P-NMR analysis was performed on an Agilent 500 spectrometer (Agilent 171 Technologies, Santa Clara, CA). In more detail, 40 mg of pre-dried lignins were dissolved in 0.5 ml of a deuterated chloroform/anhydrous pyridine solution (1:1.6, v/v) (Solvent A) and 100 μl of a solution of chromium(III) 2,4-pentanedionate (5 mg/mL) (relaxation agent) and N-hydroxy-5-norbornene-2,3-dicarboxylic acid imide (NHND, 18 mg/mL) (internal standard) dissolved in Solvent A. The solution was stirred until the lignins were fully dissolved. Finally, 100 μl of 2-Chloro-4,4,5,5-tetramethyl-1,2,3-dioxaphospholane (TMDP) was added dropwise. For the measurement, 128 scans and a relaxation delay of 10 sec were selected. Prior to the quantification analysis, the sharp peak corresponding to the TMDP+H_2O was shifted and assigned to 132.2 ppm. NMR data were processed with MestReNova (Version 14.0.2-26256, Mestrelab Research).

II. Tables

Table S1. Quantitative assignments of functional groups and their abundance present in OBS and OBSBM8 lignins, as determined ^{31}P-NMR analysis

**Lignin functional groups – Abundance (mmol/gr)**	**OBS**	**OBSBM8**	**Chemical shifts (δ_P)**
Aliphatic OH	1.52	2.12	145-150
Phenolic OH	3.45	3.08	137-144.5
COOH	0.08	0.07	134-136
**Total OH content**	5.05	5.27	134-150

III. Figures

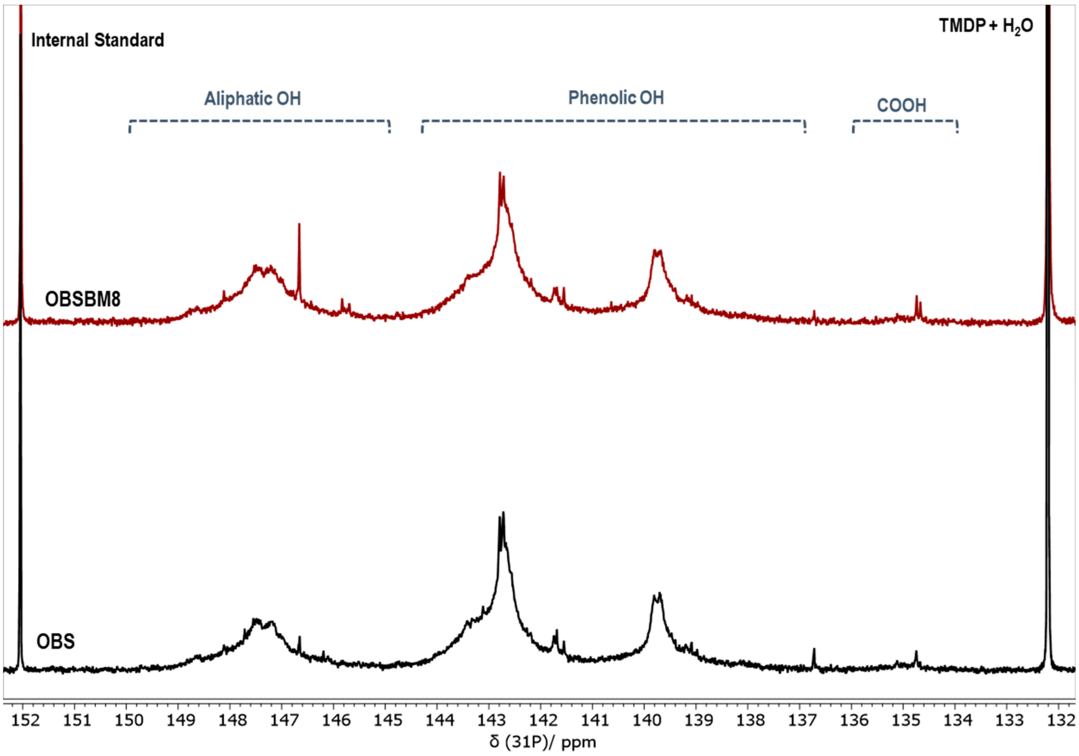

Figure S1. ^{31}P-NMR spectra of OBS and OBSBM8 lignins

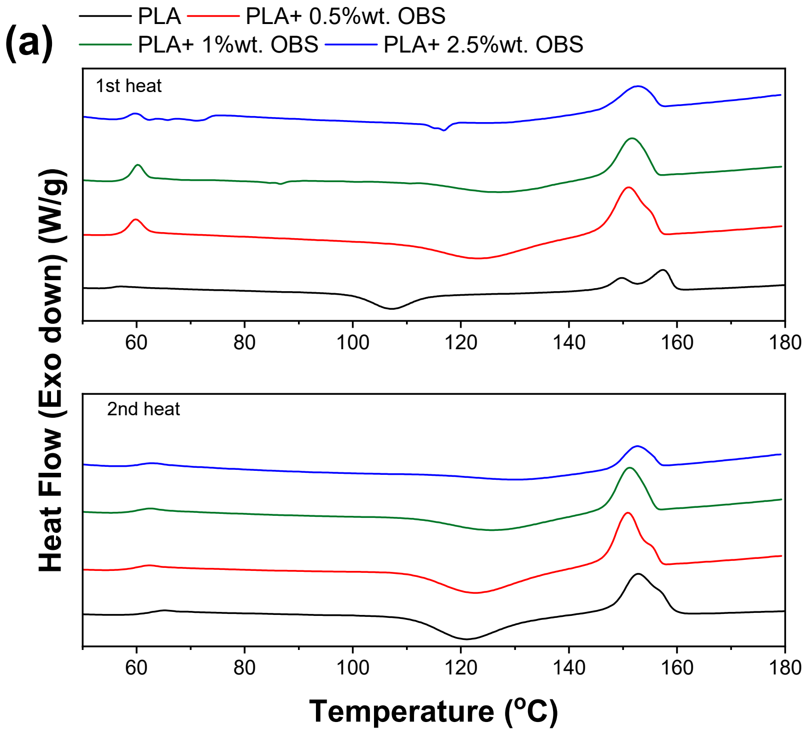

Figure S2. DSC scans of PLA/OBS composites.

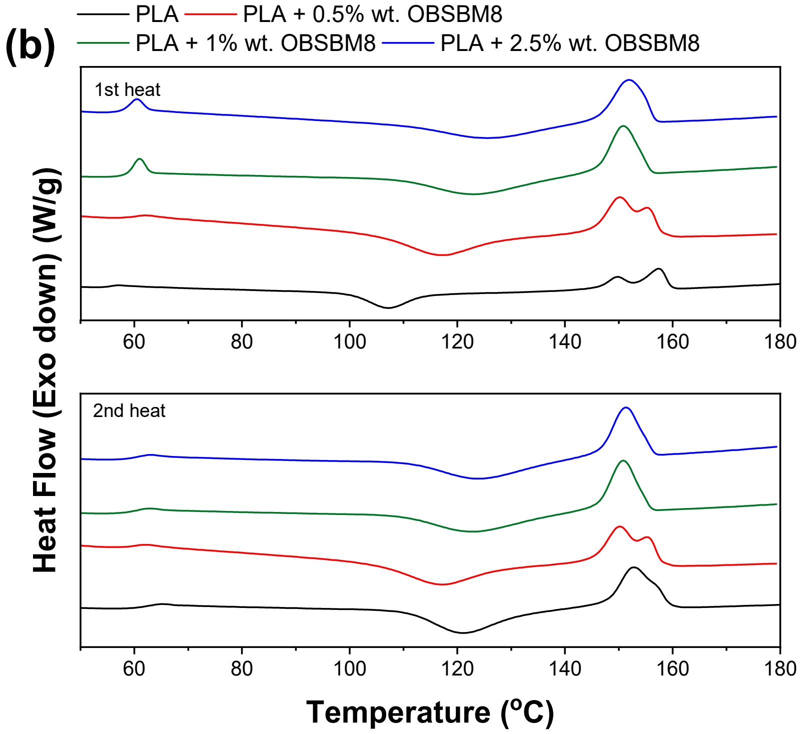

Figure S3. DSC scans of PLA/OBSBM8 composites.

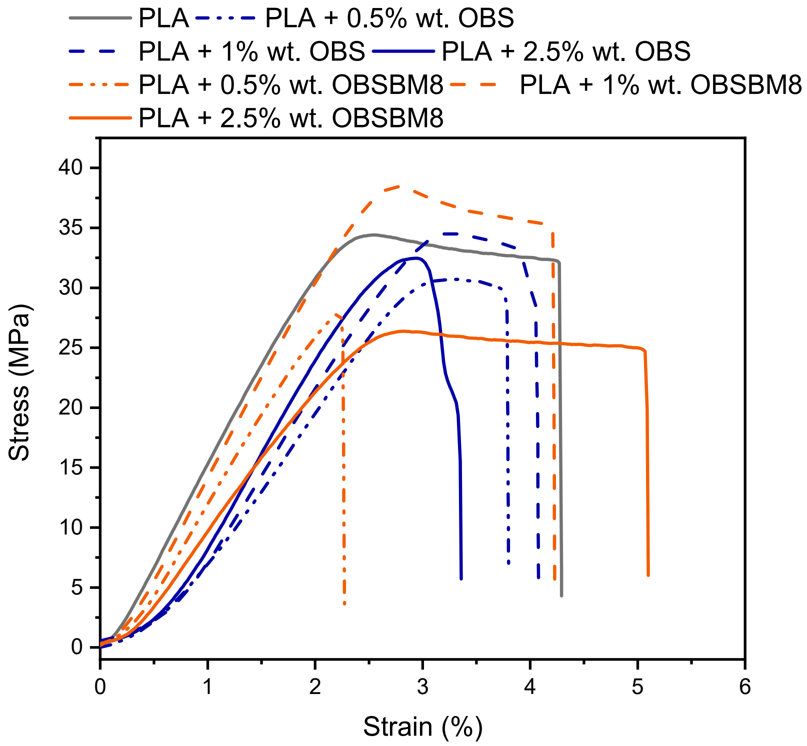

Figure S4. Indicative stress-strain curves of PLA and its composites with OBS and OBSBM8 lignin.
